# Senescence and Sexual Selection in a Pelagic Copepod

**DOI:** 10.1371/journal.pone.0018870

**Published:** 2011-04-14

**Authors:** Sara Ceballos, Thomas Kiørboe

**Affiliations:** National Institute of Aquatic Resources, Technical University of Denmark, Charlottenlund, Denmark; Institute of Marine Research, Norway

## Abstract

The ecology of senescence in marine zooplankton is not well known. Here we demonstrate senescence effects in the marine copepod *Oithona davisae* and show how sex and sexual selection accelerate the rate of ageing in the males. We show that adult mortality increases and male mating capacity and female fertility decrease with age and that the deterioration in reproductive performance is faster for males. Males have a limited mating capacity because they can fertilize < 2 females day^−1^ and their reproductive life span is 10 days on average. High female encounter rates in nature (>10 day^−1^), a rapid age-dependent decline in female fertility, and a high mortality cost of mating in males are conducive to the development of male choosiness. In our experiments males in fact show a preference for mating with young females that are 3 times more fertile than 30-day old females. We argue that this may lead to severe male-male competition for young virgin females and a trade-off that favours investment in mate finding over maintenance. In nature, mate finding leads to a further elevated mortality of males, because these swim rapidly in their search for attractive partners, further relaxing fitness benefits of maintenance investments. We show that females have a short reproductive period compared to their average longevity but virgin females stay fertile for most of their life. We interpret this as an adaptation to a shortage of males, because a long life increases the chance of fertilization and/or of finding a high quality partner. The very long post reproductive life that many females experience is thus a secondary effect of such an adaptation.

## Introduction

Marine pelagic copepods are possibly the most abundant metazoans on earth [1] and they play a major role in ocean ecosystems [2]. Consequently, much is known about their biology and ecology [3], but the ecology of ageing and its effects on individual fitness and on the evolution of life-history strategies have been very little studied. In fact ecology of ageing is not well-understood in any organism,

Ageing (or senescence) is an age-dependent reduction in survival and performance and is due to an intrinsic deterioration caused in part by increasing telomere losses and accumulation of free radicals [4]. Ageing can be partly countered by expensive repair processes at the cost of investment in, e.g., growth, sexual products and mate finding, and the optimal investment in repair and consequent rate of ageing is the result of the tradeoffs between costs and gains [recent reviews: 5, 6, 4].

Ageing can have important effects on individual fitness because of its influence on feeding rates [7], mortality rates [8], [9] and reproductive success through reduction of fecundity [10] and mating ability [11]. Some of these effects have also been demonstrated in pelagic copepods, i.e., a reduction in egg production, egg hatching success, and feeding rates with age in some species [12], [13].

Senescence can also influences individual fitness by its effects on sexual selection. Mating preferences [14], competition for mates [15], [16], [17], and the degree of mate choosiness [18] may all be age-dependent. Some models show that animals should mate preferentially with old partners because they have proven their ability to survive [e.g. 19, 20, 21] and female preference for old males has in fact been demonstrated in several groups [e.g. 22, 23, 24, 25]. Similarly, some life history optimization models predict that reproductive effort should increase towards the end of life to avoid ‘wasting’ reproductive products at death [26]. However, older individuals do not necessarily have better genes and typically are less – not more – fertile than younger ones [27], [28], as also found for female copepods [13]. In mating systems where the male only provides sperm (such as in marine copepods) female preferences for younger males may evolve, and male preference for young females has been also found in several groups, e.g. in insects [29], [30]. At the same time, choosiness may decrease during the reproductive life span because the reproductive value decreases with age [14], [31], [18].

Pelagic copepods may be a good model for sexual selection studies. Copepods have different reproductive strategies because the capacity to store sperm varies between species implying multiple and single mating systems. They also show a variety of apparent courtship behaviours that together with observations of females resistance and mate guarding and the possibility of sperm competition may be related with sexual selection processes [32] explaining why mating is far from random in these animals [33]. Male search swimming behaviour and female signalling by pheromones or hydromechanical cues ensure high mate encounter rates during most of the year [34], which would favour the development of mate selection. Spermatophore production is limited in the few species where male reproductive rates have been studied [35], [33], and sex ratios are typically female biased [36] making mating preferences in males feasible. In fact, sexual selection through mate choice has been demonstrated in females as well as males of the copepod *Acartia tonsa* [33]. Moreover, copepod males develop faster, live shorter and are generally smaller than females [37], [38]. Hence, males and females have different life histories, which may be influenced by different reproductive strategies and sexual selection process.

Here we study the effect of ageing on several parameters related to individual fitness such as mortality, mating ability, reproductive rates, attractiveness and mate choice in the marine copepod *Oithona davisae*. We focus our study on males since their ecology has been almost neglected up to now. We show that males age much faster than females and argue that this is due mainly to severe male-male competition for young, fertile females.

## Methods

### Experimental animals and general incubation methodology

*Oithona davisae* is a small (0.3 mm prosome length), ambush feeding copepod that is very common in coastal areas of temperate areas. Average adult densities can reach >10^2^ ind. L^−1^, and the sex-ratio is strongly female biased [39], [40]. Females produce a pheromone signal that rapidly search-swimming males use to locate and track down females [41], [35]. During tracking as well as precopula, where the male is attached to the female by means of his first antenna, the female attempts to escape the male and often succeeds. If successful, the male transfers a tiny (ca. 25 µm) spermatophore to each of the female's two genital pores. Females produce relatively few, large eggs that are carried in sacs until hatching [39]. Male reproductive rates are unknown.

Experimental animals came from continuous laboratory cultures. Unless otherwise noted, we conducted all the experiments with virgin adults that had matured within 24 h. These were obtained by isolating late copepodites (juveniles) individually in multi dishes (3 ml). These were kept at a natural photoperiod and 20°C, and fed the motile heterotrophic dinoflagellate *Oxyrrhis marina* (equivalent spherical diameter: 18 µm) at a saturating concentration (>500 µg carbon L^−1^). We inspected the dishes daily to get freshly moulted virgin adults. Groups of adults and individual males were incubated in 10 ml dishes. As far as we know, mating behaviour of small copepods is not affected by small incubation volumes [42], [43]. We incubated individual females in 4 ml dishes for nauplii production measurements.

### Experiments

#### Adult longevity and age-dependent mortality

We quantified the survival of copepods, both in the presence and absence of sex partners. In a first experiment, we incubated virgin adults in groups of four individuals, either males or females separately (total of 210 males and 183 females; ‘virgin treatment’), or in mixed groups of two males + two females (N =  114 of each sex, ‘mated treatment’). In a second experiment, we incubated individual males either with 10 females (a mix of different ages and reproductive status] that were replaced every 4 days, or alone (25 incubations per treatment). Every second day we fed the copepods and removed dead animals; the incubations continued until all individuals had died. We also included the longevity of 36 females obtained in the fertile life experiment (see below).

#### Male reproductive rate and reproductive life span

To measure male mating rate we incubated 20 virgin males individually, each with 10 virgin females. The incubations lasted 4 days and all females were replaced daily by fresh virgins. The females remove or lose the tiny spermatophores soon after mating and the number of successful male matings was indirectly estimated by the number of females that produced nauplii. Thus, the replaced females were individually transferred to dishes and checked for hatched eggs (nauplii) during the following 8 days. Given the results of this experiment (average mating rate was <3 matings per day) we ran the subsequent incubations with just five females.

To estimate the reproductive lifespan of males, we incubated individual virgin males with five virgin females for 24 hours and then isolated the males. They were again offered five virgin females for 24 h, either 1) at the age of 10 and 20 days (first trial, N = 24), or 2) at age 5, 7 and 10 days (second trial, N = 20). In a second experiment, we studied the affect of mating history on male mating capacity. We kept 20 virgin males isolated from females for 5, 7 or 10 days. The males were then individually incubated with 5 virgin females during 24 h and subsequently isolated. The same males were finally again offering females following the same sequence as above. Females were subsequently transferred individually to dishes to check for nauplii production.

#### Female fertile life span and offspring production

We incubated couples of virgin adults for 24 hours to get mated females. To estimate the duration of the fertile life span, 36 mated females were then incubated individually and their egg sac and nauplii production monitored daily until the female died. To examine the effect of age on the duration of fertile life a second group of virgin females that had matured 30 days earlier were incubated individually with 4 males (to increase the chance of successful matings) during 24 hours; egg sac and nauplii production was subsequently monitored until female death, as above.

#### Female fertilization needs

Mating needs of females can have a significant effect on the strength of sexual selection [44]. It is assumed that *O. davisae* females can fertilize all the eggs they can produce in their life following just one mating [45]. To test this assumption, we compared offspring production of females that had mated once (the above experiment) with females that had been offered males repeatedly. Individual females were incubated for 24 hours with four new males every 4 days. Nauplii production was followed until egg production ceased, as above.

#### Effect of age and duration of celibacy on the degree of choosiness

Copepods have sequential mate choice using a threshold rule (whether the mating criterion is fixed or flexible is not solved yet) and therefore couple incubations can be used for mate choice trials [33]. To examine if choosiness changes with age, 20 couples of different age after maturation (1, 3, 5, 7, 10, 20, 30 and 40 days) were incubated for 24 hours. The response variable was the fraction of couples that had mated during this period assuming that this fraction is a proxy of the degree of choosiness [33]. Mated couples were identified as those where the female subsequently produced nauplii (individual incubation for 8 days).

#### Age as a trait for mate selection

To examine if animals choose partner based on age, virgin copepods were incubated for 24 hours in couples combining old and young adults. We had four treatments: 1) young female and young male (YY), 2) young female and old male (YO), 3) old female and young male (OY), and 4) old female and male (OO). We used animals from several cohorts to get different ages simultaneously. Given our survival observations (see results), ‘old females’ were defined as 30 days post maturation and ‘old males’ as 10–12 days post maturation. N =  20 for each treatment.

### Data analysis

We used ANOVA, Kruskal-Wallis ANOVA, t-tests and Mann-Whitney Rank Sum test to compare means, Chi-square tests to examine differences between frequencies and correlation and regression analysis for testing relationships between variables; when the dependent variable was the mating frequency a logistic regression was used [46]. Survival analyses were used for life span comparisons [47]. Data were checked for normality distribution and homogeneity of variances where appropriate. Probability tests were two-tailed. The Bonferroni's method was used for multiple pair comparisons. Statistical analyses were performed by means of Sigma Stat 3.5 and SPSS 18 (for survival analyses). Data are shown as mean ±95% confidence and standard deviation. α = 0.05.

## Results

### Adult lifespan and mortality

**Figure 1:**
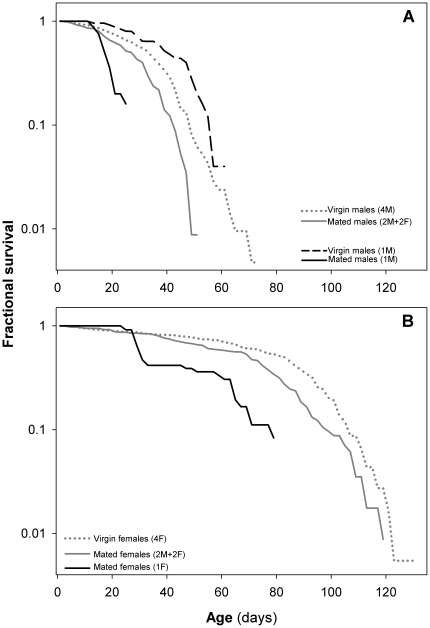
Male (A) and female (B) life span. Copepods were incubated individually or in groups in two different ways, 1) in celibacy (‘virgin copepods’) or with access to the other sex (‘mated copepods’), see methods for more details. Overall comparison for males; Mantel-Cox test: χ^2^ = 65.5, df = 1, p<0.01. The pair comparison of virgin males incubated individually or in groups was not significant; Mantel-Cox test: χ^2^ = 5.6, df = 1, p = 0.05, (corrected α = 0.008). Overall comparison for females; Mantel-Cox test: χ^2^ = 58.1, df = 2, p<0.01.

The average longevity of virgin males was significantly longer than that of mated males in both experiments (Fig. 1A). The effect was most pronounced in the experiment where one male was incubated with 10 females [40 (95% CI: 35–45) vs. 19 (17–21) days] but still significant in the incubation with only 2 females per 2 males [31(29–33) and 26 (24–28 days]. The life span of males in celibate was the same irrespective of the density in the incubations (single vs. group of 4). The maximum individual lifespan of mated and virgin males were between 1 and 2 months and > 3 months, respectively (Fig. 1A). Thus, there is a significant mortality penalty on sexual activity in males.

As for females, longevity of virgin females was significantly longer than that of mated females (Fig. 1B). Virgin females lived an average of 73 (68–77) days, the longevity of females incubated in groups of 2 males and 2 females was 64 (58–69) days, and females that had access to different groups of males lived 43 (37–50) days. Hence, mating activity also had an effect on female longevity although not as strong as in the males.

Irrespective of treatment and gender, all survival curves are convex when plotted on a log-scale (Fig. 1A, B). This demonstrates that mortality rate increases with age (age-independent mortality rate would lead to straight lines). Thus, ageing becomes manifest as elevated mortality.

### Male mating rates and duration of reproductive life

**Figure 2:**
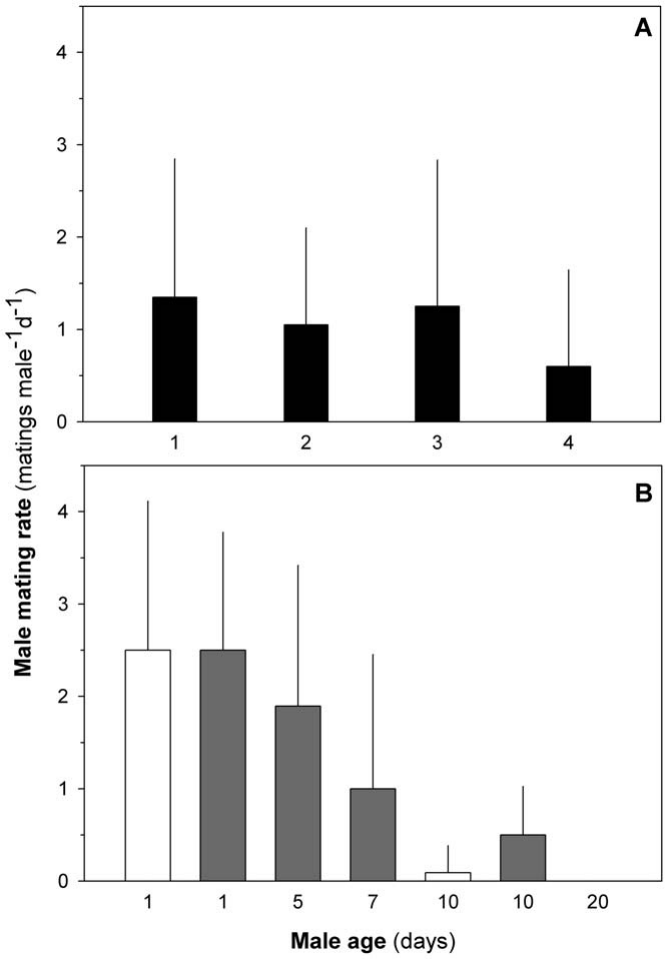
Male mating rates and reproductive lifespan. Mating rates during the first 4 days of male adult life (A). There was no significant temporal trend during this period (Repeated Measures ANOVA Test: F_19,3_ = 1.4, p>0.1). Mating rates of 1 (control), 5, 7, 10, and day old virgin males (B). Data from the first experiment (1, 10 and 30 day old males) are shown as white bars and data from the second experiment (1, 5, 7 and 10 day old males) are shown in grey. Male mating capacity significantly decreases during the first ten days of reproductive time (Repeated Measures ANOVA Test: F_23,2_ = 35.7, p<0.001). Mean ± SD. Male age is given relative to time of maturation. We used different cohorts of animals in experiments showed in panel A and B explaining the different mating rates of males younger than 5 days.

**Figure 3:**
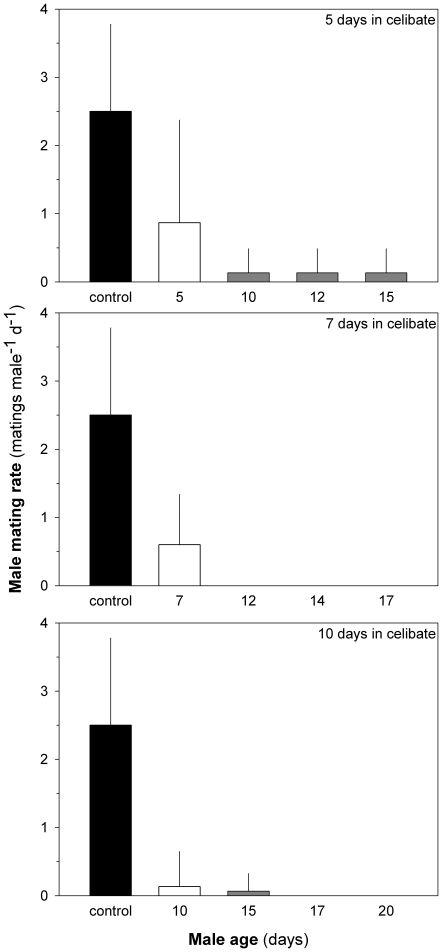
Effect of age and celibacy on male mating rates. Mating rates of males as a function of age and time in celibacy. Male ages are relative to time of maturation. Mating rate of males matured within 1 day are shown as black bars (controls), white bars refer to mating rates of males incubated with females for the first time after different periods of celibacy. These males were repeatedly exposed to females (grey bars). Time in celibacy (5, 7 or 10 days) had no effect on male mating capacity (ANOVA test: F_2,44_ = 2.0, p>0.1), but an effect of age on mating capacity is found when comparing mating rates of males in celibate with control males; 1 day old males have higher mating rates (ANOVA test: F_3,61_ = 15.9, p<0.01, Tukey post-hoc test: 1>(5 = 7 = 10). Mean ± SD.

Males have a low mating capacity. Average mating rate was <2.5 matings per day during the first 4 days of adult life (Fig. 2A), 25% of the males never mated during this period, and 50–65% of males did not mate on a daily basis. There was no significant temporal trend in mating rate during the first 4 days of male adult life (Fig. 2A). However, thereafter male mating rate declined significantly and considerably (Fig. 2B). Only 4% of 10-day-old males mated, and 20 days after maturation males did not mate at all. This senescence is independent of previous mating history since males living in celibacy for 5–10 days after maturation had similarly low mating rates when eventually offered females (Fig. 3). Hence, age has an important effect on male mating capacity and males get reproductively old within ca. 10 day after maturation. During this time they produced an average of 11(8–14) spermatophores, which means that they are able to mate with 5.5 (4–8) females (2 spermatophores transferred in each mating event).

Male mating history had only a slight and mainly insignificant effect on current reproductive effort *per se* (table 1), but the intensity of reproduction impacted male longevity: 92% of the males that were exposed to females only once survived to the age of 10 days, whereas only 50% of the males that were exposed to females twice survived to this age (data from the male fertile life experiment; χ2 = 7.6, df = 1, p<0.01).

**Table 1:** Mating history effects on male mating capacity.

Male age (days)	Number of previous mating oportunities	Mating rate	Statisitical test
5	0 vs. 1	0.9 vs. 1.9	U = 202.0 P = 0.03
7	0 vs. 1	0.6 vs. 1.0	U = 151.5 P>0.1
19	0 vs. 1 vs. 3	0.1 vs. 0.1 vs. 0.2	H = 2.3 df = 2 p>0.1
14–15	1 vs. 2 vs. 3	0.1 vs. 0.0 vs. 0.1	H = 2.1 df = 2 p>0.1

The mating rates of males (matings male^−1^ day^−1^) with different mating histories. Mating rates are compared between males of the same age with 0, 1, 2 or 3 previous mating opportunities. Data from male reproductive life experiments (see Figs. 2B and 3).

### Female duration of fertile life

**Figure 4:**
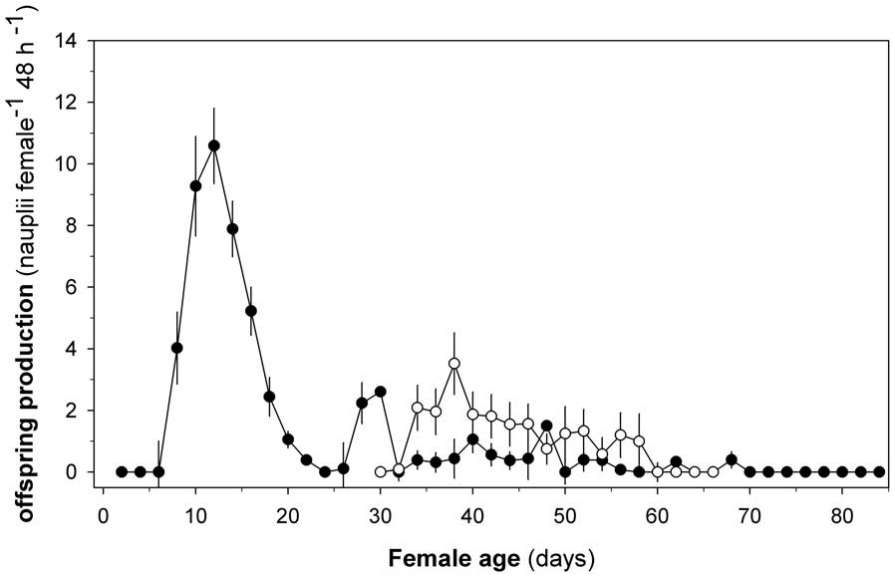
Female fertile life span. The offspring production dynamics subsequent to mating in recently matured females (black dots) and in females that had matured 30 days earlier (white dots). The produced number of nauplii per female during 2-day periods is reported as mean ± SEM. The offspring production was lower in old females (t-test: t_55_ = 5.5, p<0.01), but both old and young females have a similar fertile lifespan (t-test: t_55_ = 2.0, p = 0.049). Female age is relative to time of maturation.

Females that were fertilized immediately following maturation produced fertile eggs for an average of 16±11days. Females produced most of the fertile eggs during their first two weeks of adult life with a peak 10 days after maturation, after which the decrease in production is significant (r = −0.6, p<0.01) (Fig. 4). Females produced just 5±2 batches of eggs resulting in 49 nauplii ±19 nauplii following one mating (Table 2). Total number of egg batches and fertile eggs produced were not related to the total life span of the individual female (r = 0.04 and r = 0.2, p>0.1 for both cases) and the females lived long after they ceased producing fertile eggs (Fig. 1B).

**Table 2:** Effect of multiple mating on reproductive output of females.

Reproductive parameter	One mating	Repeated matings	Statisitical test
Number of egg batches produced	4.7 (4.0–5.4)	4.3 (3.3–5.4)	U = 420.5 p>0.1
Number of nauplii produced	49.1 (42.7–55.5)	48.9 (35.7–62.0)	U = 404.5 p>0.1
Duration of fertile period (days)	16.2 (12.6–19.8)	12.3 (8.5–16.1)	t_56_ = 1.5 p>0.1

Reproductive parameters of females exposed to one male just after maturation (one mating) and in females that had the option of mating several times during their life span (repeated mating). Mean ±95% CI.

We wondered whether the relatively short fertile period of the females relative to their total life span was due to exhaustion of egg production capacity or due to an age-dependent loss of fertility. We therefore examined fertility in old virgins (30 days after maturation). Following mating, these females produced fertile eggs in a temporal pattern resembling that of the younger females, with a peak 8 days after mating and a subsequent gradual decline, but at a significantly lower level (Fig. 4). Seventy five percent of the old virgin females mated, but 1/3 of these did not produce any nauplii even though they had spermatophores attached. The offspring production was 18±23 nauplii (95% CI: 8–29), ca. 3 times less that of the young females, but their fertile life lasted almost as long that of the young females (11±10 days, 95% CI: 6–15, Fig. 4). These observations suggest a rather fixed duration of the fertile period independent of the age at which the female is fertilized, but a lower fertility of older females.

### Female fertilization needs

Females can fertilize all the eggs they produce with just one mating. The number of egg batches, total number of offspring and the duration of fertile life for females repeatedly exposed to males were not different from that of females exposed to males only once (Table 2).

### Age effects on choosiness and mate choice

**Figure 5:**
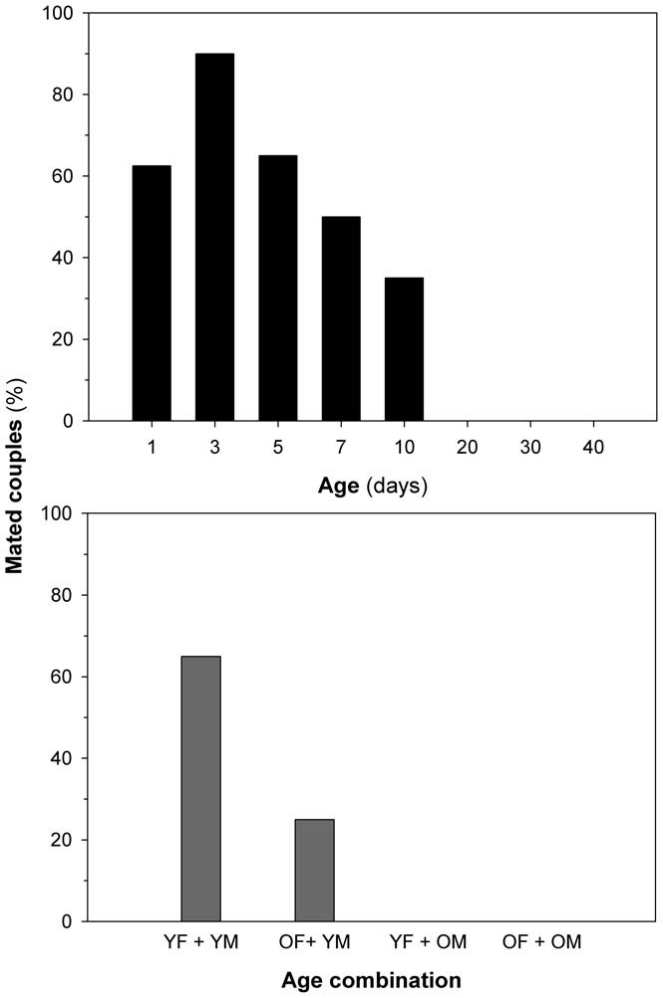
Copepod age effects on mate selectivity. The percentage of couples that mate in 24-h incubations as a function of age (A). The fraction of mated couples is taken as a proxy of choosiness. Age has an effect on whether couples mated [logistic regression; Likelihood ratio test: χ^2^ = 85.4, df = 1, p<0.01, Hosmer-Lemeshow test: χ^2^ = 10.7, df = 1, p>0.05, Wald test for age: χ^2^ = 30.3, df = 1, p<0.01). Mating success in even-aged and odd-aged couples (B). F =  female, M =  male, Y =  young and O =  old, see methods. Mating success is higher for young copepods (we compare YY couples versus OY ones for females; Chi-square test: χ^2^ = 4.95, df = 1, p<0.05, and YY couples versus YO ones for males: χ^2^ = 16.4, df = 1, p<0.01).

Age has an effect on whether virgin couples mate. Among couples formed by copepods of the same age, mating success initially increased, although insignificantly, suggesting a decrease in choosiness with duration of celibacy. Subsequently, mating success decreased significantly with couple age, suggesting senescence effect (Fig. 5A). Couples older than 10 days did not mate at all.

Among couples combining old and young copepods, young males apparently preferred young to old females, as indicated by a higher mating success (Fig. 5B). Similarly, young females apparently prefer young to old males. This latter result may be also due to the loss of mating capability in old males, cf. above.

## Discussion

Pelagic copepods age in the sense that several aspects of their performance decline with age. Ageing effects on egg production, hatching success and feeding rates have previously been demonstrated for female copepods [13], but we have here extended senescence effects to additional aspects related to the individual fitness in *O. davisae*. We show that mortality increases and reproductive performance decreases with age in both males and females and most so for males, that sexual activity accelerates senescence, again most pronounced for males, and finally that females may have a long post-reproductive life.

Acceleration of ageing processes in males due to mating has been demonstrated in other taxa [48]. For instance, mating leads to suppression of the male immune system in insects [49]. Among pelagic copepods, males are generally the weaker sex: they have a higher mortality rate than females even in the absence of predators and sexual activity ([38] and Fig. 1) and they are more susceptible to infections [50] and to harmful effects of dinoflagellate toxins [51]. The difference between male and female adult longevity in *O. davisae* in the laboratory (20–40 days versus 40–70 days, Fig. 1) would suggest adult male to female sex ratios in the field of about 1∶2 if ‘physiological death’ was the only source of mortality. Actual male-to-female sex ratios in the field are much more female biased in *O. davisae* (∼ 1∶10; [39]), and in many other species [36] suggesting that other sources of mortality are also higher in males than in females. An important difference is the much higher swimming speed and consequent predator encounter rates in males than in females [52]. While the motility of the ambush feeding *O. davisae* females is very low, the males often sacrifice feeding and swim rapidly in order to search for females leading to higher predator encounter rates in males than in females [35].

The difference in ageing rates between males and females is in agreement with predictions from life history theory and trade-offs between investment in reproduction and somatic maintenance. Organisms that suffer from high extrinsic mortality should invest more in reproduction and less in maintenance and, thus, should age faster and be more susceptible to diseases [6]. The significant survival penalty of mating in males reinforces gender differences. Hence males may gain more fitness benefits by sacrificing viability for sexual performance, whereas females may gain more benefits by investing in longevity [53]. Thus, *O. davisae* males seem to have a ‘live fast and die young’ strategy with low investment in maintenance, which is also consistent with reports of a higher levels of oxidative stress in male than in female copepods [13]. This strategy is quite common in males of different taxa, but up till now it has not been proposed for copepods.

Why do *O. davisae* males have a ‘live fast and die young’ strategy? We propose that the high investment in mate searching and the consequent high mortality and rapid senescence may be explained in the context of sexual selection, male-choice and male-male competition for high quality females. There are three necessary conditions for males to develop mate choosiness: there should be a significant cost to mating, mate encounter rate should be high, and there should be a significant variation in the quality of potential mates that can be perceived by the males [54]. First, mating is in fact costly to males in terms of a significant mortality penalty and a loss of future mating opportunities due to the limited mating rate. Secondly, because of the very female-biased adult sex ratios observed in the field, mate encounter rates are ca. 10 times higher for males than for females, which favours male choosiness. At typical summer population densities, males encounter females at rates that are very much higher than the rate at which they can mate, up to 10^3^ females male^−1^ day^−1^, with an annual average exceeding 10 females male^−1^d^−1^ in the field population examined by Uye & Sano [39], [35]. Finally, the ‘quality’ of females varies significantly as the fecundity of virgins declines rapidly with age: mating with a recently matured rather than with a 30 day old virgin female implies a 3-fold difference offspring production following a mating. Males may also mate preferentially with young females because the probability that the female is virgin declines with age. Although females may occasionally mate several times (personal observations), *O. davisae* females can fertilize all their eggs in one mating as we show here, and it is believed that sperm produced by males that mate first have priority for fertilization [41]. Copepod males seem able to discriminate between females based on their reproductive status [55] in addition to age, but the mechanism is not understood. It may be that this information is carried in the pheromone signal that the female produces or, in the case of reproductive status, that mated females produce little or no pheromones. Despite the lack of a known mechanism for assessing female ‘quality’, these considerations are all consistent with the observed male preference for young females. Further, because females do not stay forever young, a race between males to encounter still-virgin young females may develop between the males, possibly explaining the unusually high investment in mate searching that is found in males of this species. A high male mortality rate is common in mating systems with a high male-male competition for the access to females [56], [57].

The residual reproductive value decreases with animal age so older animals should become less selective when choosing a mate [18], [58]. That should be more pronounced for virgin animals, because the risk of remaining unmated increases with age. However, we observed only an insignificant increase in mating success (as a proxy for decreased choosiness) in our couples of virgin copepods with time (Fig. 2A). Any effect may have been overridden by ageing effects because *O. davisae* males rapidly lose mating performance (Fig. 2B). For the same reason female preference against old males are inconclusive (Fig. 5B). Females can choose young partners as a way of increasing fertility and/or having fitter offspring [59], [60], [61], [62]. The copepod female mating preferences based on male age deserves future attention.

Females of *O. davisae* age at a much slower rate than the males and they have a rather short and apparently fixed reproductive period that is nearly independent of age. The short reproductive period of *O. davisae* females (around 2 weeks) is even more surprising in light of their longevity and the consequent extended period of post-reproductive life. Of course longevity and post-reproductive life are shorter in the field due to predation, or food limitation although females of the *Oithona* genus appear to have very low mortalities relative to other small copepods, even in the field [63]. Interestingly, the duration of the reproductive period relative to the average longevity in other small copepods is substantially longer, e.g., 26 day reproductive period in *Acartia tonsa* [64] and 22 days in *Centropages typicus* [65] relative to average longevities of less than 30 days for both species under lab conditions (own unpublished data). What are the fitness benefits of investment in maintenance and long life rather than reproductive investment in the females that lead to rather low reproductive output and a long post-reproductive life? Evolutionary theory predicts that a long post-reproductive life can evolve only if post-reproductive females can gain a fitness advantage by improving the reproductive success of their offspring by means of some kind of care (the ‘grandmother hypothesis’; [66]), but that is obviously not applicable to copepods. We suggest that the capability of virgin females to remain fertile even at high age may be interpreted as an adaptation to fertilization limitation owing to low male encounter rates during long periods of the year and/or low mating capability of the males. It could also increase their chance of finding and choosing a high quality partner without a large loss in offspring production. A long post-reproductive life of those individuals that mate at early age would then be a secondary consequence of such an adaptation. The fact that male:female sex ratios in field populations of *O. davisae* are about 1∶10 combined with the low mating capacity of males (on average 5 life-time matings per male in a predator-free environment) indeed suggests that a large fraction of the females in a population will never be mated and that fertilization limitation is severe, as suggested earlier [35]. Field observations that only about 1/3 of the adult females are actively reproducing support this picture [39]. However, whether this is an evolutionary stable strategy life remains to be examined.

## References

[1] Humes AG (1994). How many copepods?. Hydrobiologia 292/.

[2] Legendre L, Rivkin RB (2002). Fluxes of carbon in the upper ocean: regulation by food-web control nodes.. Mar Ecol Prog Ser.

[3] Mauchline J (1998). The biology of calanoid copepods..

[4] Ricklefs RE (2008). The evolution of senescence from a comparative perspective.. Funct Ecol.

[5] Kirkwood TB, Austad SN (2000). Why do we age?. Nature.

[6] Kirkwood TB (2002). Evolution of ageing.. Mech Ageing Dev.

[7] Catry P, Phillips RA, Phaln B, Croxall JP (2006). Senescence effects in an extremely long-lived bird: the grey-headed albatross *Thalassache chrysostoma*.. Proc Biol Sci.

[8] Sibly RM, Collett D, Promislow DEL, Peacock DJ, Harvey PH (1997). Mortality rates of mammals.. J Zool Lond.

[9] Bonduriansky R, Brassil CE (2002). Rapid and costly ageing in wild male flies.. Nature.

[10] Jones TM, Elgar MA (2004). The role of male age, sperm age and mating history on fecundity and fertilization success in the hide beetle.. Proc R Soc Lond B.

[11] Long CE, Markow TA, Yaeger P (1980). Relative male age, fertility, and competitive mating success in *Drosophila melanogaster*.. Behav Genet.

[12] Carlotti F, Rey C, Javanshir A, Nival S (1997). Laboratory studies on egg and faecal pellet production of *Centropages typicus*: effect of age, effect of temperature, individual variability.. J Plankton Res.

[13] Rodríguez-Graña L, Calliari D, Tiselisus P, Hasen BW, Nilsson Skold H (2010). Gender-specific ageing and non-Mendelian inheritance of oxidative damage in marine copepods.. Mar Ecol Prog Ser.

[14] Gray DA (1999). Intrinsic factors affecting female choice in house crickets: time cost, female age, nutritional condition, body size, and size-relative reproductive investment.. J Insect Behav.

[15] Hu HH, Morse DH (2004). The effect of age on encounters between male crab spiders.. Behav Ecol.

[16] Radwan J, Michalczyk Ł, Prokop Z (2005). Age dependence of male mating ability and sperm competition success in the bulb mite.. Animal Behav.

[17] Fischer K, Perlick J, Galetz T (2008). Residual reproductive value and male mating success: older males do better.. Proc R Soc Lond B.

[18] Moore PJ, Moore AJ (2001). Reproductive aging and mating: The ticking of the biological clock in the female cockroaches.. Proc Nat Acad Sci.

[19] Trivers R, Campbell B (1972). Parental investment and sexual selection.. Sexual selection and the descend of man.

[20] Andersson M (1994). Sexual selection..

[21] Kokko H, Lindström J (1996). Evolution of female preference for old mates.. Proc R Soc Lond B.

[22] Zuk M (1987). Variability in attractiveness of male field crickets (Orthoptera: Gryllidae).. Anim Behav.

[23] Zuk M (1988). Parasite load, body size, and age of wild-caught male field crickets (Orthoptera: Gryllidae): effects on sexual selection.. Evolution.

[24] Grahn M, Von Schantz T (1994). Fashion and age in pheasants: age differences in mate choice.. Proc R Soc Lond B.

[25] Simmons LW (1995). Correlates of male quality in the field cricket, *Gryllus campestris* L.: Age, size, and symmetry determine pairing success in field populations.. Behav Ecol.

[26] Isaac JL, Johanson CN (2005). Terminal reproductive effort in a marsupial.. Biol Lett.

[27] Hansen TF, Price DK (1995). Good genes and old age: do old mates provide superior genes?. J Evol Biol.

[28] Brooks R, Kemp DJ (2001). Can older males deliver the good genes?. Trends Ecol Evol.

[29] Jones TM, Balmford A, Quinnell RJ (2000). Adaptive female choice for middle-aged mates in a lekking sandfly.. Proc R Soc Lond B.

[30] Simmons LW, LLorens T, Schinzig M, Hosken D, Craig M (1994). Sperm competition for male mate choice and protandry in the bushcricket, *Requena verticalis* (Orthoptera: Tetiigonidae).. Anim Behav.

[31] Kodric-Brown A, Nicoletto PF (2001). Age and experience affect female choice in the guppy (*Poecilia reticulata*).. Am Nat.

[32] Titelman J, Varpe Ø, Eliassen S, Fifsen Ø (2007). Copepod mating: chance or choice?. J Plankon Res.

[33] Ceballos S, Kiørboe T (2010). First evidences of sexual selection by mate choice in marine zooplankton.. Oecologia.

[34] Kiørboe T, Bagøien E (2005). Motility patters and mate encounters rates in planktonic copepods.. Limnol Oceanogr.

[35] Kiørboe T (2007). Mate finding, mating, and population dynamics in a planktonic copepod *Oithona davisae*: There are too few males.. Limnol Oceanogr.

[36] Kiørboe T (2006). Sex, sex-ratio, and the dynamics of pelagic copepod populations.. Oecologia.

[37] Gilbert JJ, Willianson CE (1983). Sexual dimorphism in zooplankton (Copepoda, Cladocera, and Rotifera).. Ann Rev Ecol Syst.

[38] Hirst AG, Bonnet D, Conway DVP, Kiørboe T (2010). Does predation control adult sex-ratios and longevities in marine pelagic copepods?. Limnol Oceanogr.

[39] Uye S-H, Sano K (1995). Seasonal reproductive biology of the small cyclopoid copepod *Oithona davisae* in a temperate eutrophic inlet.. Mar Ecol Prog Ser.

[40] Hirota R (1990). Microdistribution of the marine copepod *Oithona davisae* in the shallow waters of Ariake-kai mud flats, Japan.. Mar Biol.

[41] Uchima M, Murano M (1988). Mating behaviour of the marine copepod *Oithona davisae*.. Mar Biol.

[42] Choi K-H, Kimmerer WJ (2008). Mate limitation in an estuarine population of copepods.. Limnol Oceanogr.

[43] Choi K-H, Kimmerer WJ (2009). Mating success and its consequences for population growth in an estuarine copepod.. Mar Ecol Prog Ser.

[44] Clutton-Brock TH (1988). Reproductive success: Studies of individual variation in contrasting breeding systems..

[45] Uchima M (1985). Copulation in the marine copepod *Oithona davisae* Ferrari & Orsi I. Mate discrimination.. Bull Plankton Soc Japan.

[46] Agresti A (2002). Categorical Data Analysis..

[47] Elandt-Johnson R, Johnson N (1980). Survival Models and Data Analysis..

[48] Bonduriansky R, Brassil CE (2005). Reproductive ageing and sexual selection on male body size in a wild population of antler flies (*Protopiophila litigara*).. J Evol Biol.

[49] McKean KA, Nunney L (2001). Increased sexual activity reduces male immune function in *Drosophila melanogaster*.. Proc Nat Acad Sci.

[50] Wedekind C, Jakobsen PJ (1998). Male-biased susceptibility to helminth infection: an experimental test with a copepod.. Oikos.

[51] Avery DE, Altland KK, Dam HG (2008). Sex-related differential mortality of a marine copepod exposed to a toxic dinoflagellate.. Limnol Oceanogr.

[52] Kiørboe T (2008). Optimal swimming strategies in mate-searching pelagic copepods.. Oecologia.

[53] Bonduriansky R, Maklakov A, Zajitschek F, Brooks R (2008). Sexual selection, sexual conflict and the evolution of aging and life span.. Fuct Ecol.

[54] Kokko H, Monaghan P (2001). Predicting the direction of sexual selection.. Ecol Lett.

[55] Kelly LS, Snell TW, Lonsdale DJ (1998). Chemical communication during mating of the harpacticoid *Trigriopus japonicus*.. Phil Trans R Soc Lond B.

[56] Tufvesson MB, Tufvesson T, von Schantz KJ, Wilhelmson M (1999). Selection for sexual male characters and their effects on other fitness related traits in white leghorn chickens.. J Anim Breed Gen.

[57] McElligott AG, Hayden TJ (2000). Lifetime mating success, sexual slection, and life history of fallow bucks (*Dama dama*).. Behav Ecol Sociobiol.

[58] Kodric-Brown A, Nicolleto PF (2001). Age and experience affect female choice in the guppy (*Poecilia reticulata*).. Am Nat.

[59] Radwan J (2003). Male age, germline mutations and the benefits of polyandry.. Ecol Lett.

[60] Prokop ZM, Stuglik M, Żabi'nska I, Radwan J (2007). Male age, mating probability, and progeny fitness in the buld mite.. Behav Ecol.

[61] Price KP, Hansen TF (1998). How does offspring quality change with age in male *Drosophila melanogaster*.. Behav Gen.

[62] Serre V, Robaire B (1998). Paternal age affects fertility and progeny outcome in the brown Norway rat.. Fertil Steril.

[63] Eiane K, Ohman MD (2004). Stage-specific mortality of *Calanus finmarchicus*, *Pseudocalanus elongatus* and *Oithona similis* on Fladen Ground, North Sea, during a spring bloom.. Mar Ecol Prog Ser.

[64] Parrish KK, Wilson DF (1978). Fecundity studies on *Acartia tonsa* (Copepoda: Calanoida) in standardized culture.. Mar Biol.

[65] Carlotti F, Rey C, Javanshir A, Nival S (1997). Laboratory studies on egg and faecal pellet production of *Centropages typicus*: effect of age, effect of temperature, individual variability.. J Plankton Res.

[66] Landenperä M, Lummaa V, Helle S, Tremblay M, Russell AF (2004). Fitness benefits of prolonged post-reproductive lifespan in women.. Nature.

